# Olfactory ecto-mesenchymal stem cell-derived exosomes ameliorate murine Sjögren’s syndrome by modulating the function of myeloid-derived suppressor cells

**DOI:** 10.1038/s41423-020-00587-3

**Published:** 2021-01-06

**Authors:** Ke Rui, Yue Hong, Qiugang Zhu, Xiaofei Shi, Fan Xiao, Hailong Fu, Qing Yin, Yida Xing, Xinfeng Wu, Xiaodan Kong, Huaxi Xu, Jie Tian, Shengjun Wang, Liwei Lu

**Affiliations:** 1 Department of Laboratory Medicine, Affiliated Hospital of Jiangsu University, Zhenjiang, China; 2 Department of Immunology, Jiangsu Key Laboratory of Laboratory Medicine, School of Medicine, Jiangsu University, Zhenjiang, China; 3 Department of Rheumatology, The First Affiliated Hospital and College of Clinical Medicine, Henan University of Science and Technology, Luoyang, China; 4 Department of Pathology and Shenzhen Institute of Research and Innovation, The University of Hong Kong; Chongqing International Institute for Immunology, Hong Kong, China; 5 Center for Clinical Laboratory, The First Affiliated Hospital of Soochow University, Suzhou, China; 6 Department of Rheumatology, The Second Affiliated Hospital of Dalian Medical University, Liaoning, China

**Keywords:** mesenchymal stem cells, exosomes, MDSCs, Sjögren’s syndrome, autoimmune diseases, Autoimmunity, Immunosuppression

## Abstract

Sjögren’s syndrome (SS) is a systemic autoimmune disease characterized by progressive inflammation and tissue damage in salivary glands and lacrimal glands. Our previous studies showed that myeloid-derived suppressor cells (MDSCs) exhibited impaired immunosuppressive function during disease progression in patients with SS and mice with experimental Sjögren’s syndrome (ESS), but it remains unclear whether restoring the function of MDSCs can effectively ameliorate the development of ESS. In this study, we found that murine olfactory ecto-mesenchymal stem cell-derived exosomes (OE-MSC-Exos) significantly enhanced the suppressive function of MDSCs by upregulating arginase expression and increasing ROS and NO levels. Moreover, treatment with OE-MSC-Exos via intravenous injection markedly attenuated disease progression and restored MDSC function in ESS mice. Mechanistically, OE-MSC-Exo-secreted IL-6 activated the Jak2/Stat3 pathway in MDSCs. In addition, the abundant S100A4 in OE-MSC-Exos acted as a key factor in mediating the endogenous production of IL-6 by MDSCs via TLR4 signaling, indicating an autocrine pathway of MDSC functional modulation by IL-6. Taken together, our results demonstrated that OE-MSC-Exos possess therapeutic potential to attenuate ESS progression by enhancing the immunosuppressive function of MDSCs, possibly constituting a new strategy for the treatment of Sjögren’s syndrome and other autoimmune diseases.

## Introduction

Sjögren’s syndrome (SS) is characterized by progressive inflammation and tissue damage in salivary glands (SGs) and lacrimal glands.^1^ Although much progress has been made in elucidating the pathophysiological mechanisms underlying SS, the etiology of this disease is still unclear. It has been reported that SS is associated with an imbalance of T helper (Th1)/Th2 cells, but recent studies have demonstrated that Th17 cells, T follicular helper cells, and T follicular regulatory cells also participate in the development of SS.^2,3^ As a histopathological hallmark of SS, various types of immune cells infiltrate SG tissue and secrete many inflammatory cytokines, such as IL-1β, IFN-γ, and TNF-α, leading to tissue destruction and functional loss of the SG.^4^

Myeloid-derived suppressor cells (MDSCs) have been identified as a heterogeneous population of immature myeloid cells with immunosuppressive function.^5^ MDSCs are characterized by coexpression of Gr-1 and CD11b and are further subdivided into two discrete subpopulations: polymorphonuclear MDSCs (PMN-MDSCs) with a CD11b^+^Ly-6G^+^Ly-6C^lo^ phenotype and monocytic MDSCs (M-MDSCs) with a CD11b^+^Ly-6G^-^Ly-6C^hi^ phenotype. PMN-MDSCs produce high levels of reactive oxygen species (ROS) and arginase-1, whereas M-MDSCs are characterized by their production of high levels of nitric oxide (NO). MDSCs play a critical role in tumor immune escape mechanisms because of their function in regulating T cell-mediated antitumor immunity.^6,7^ Accumulating evidence has indicated the involvement of MDSCs in the pathogenesis of various autoimmune disorders. Our recent studies reported a pivotal role of MDSCs in the development of SS.^8,9^ We found that the MDSC population was significantly increased in mice with experimental Sjögren’s syndrome (ESS), but their suppressive function decreased gradually with the progression of the disease. Therefore, it remains to be established whether maintaining or restoring the suppressive function of MDSCs has therapeutic implications in SS.

Many animal and clinical studies have demonstrated the therapeutic potential of mesenchymal stem cells (MSCs) in ameliorating various disorders, including autoimmune diseases.^10^ Recently, exosomes have been described as essential paracrine products of MSCs, and they may constitute a replacement for cell-based therapy.^11^ In particular, MSC-derived exosomes (MSC-Exos) exert strong effects on tissue repair and perform an immunomodulatory function in treating inflammatory diseases.^12^ Exosomes are nanosized membrane vesicles that function as an important communication tool among various cell types.^13^ Exosomes carry various biological molecules, including proteins, lipids and RNAs, and then transfer their cargo to recipient cells, functioning as extracellular messengers to mediate intercellular communication.^14,15^

Olfactory ecto-mesenchymal stem cells (OE-MSCs), a new type of resident stem cells in the olfactory lamina propria, have a high proliferation rate, self-renewal capability, and multilineage differentiation potential.^16–18^ In addition to their potential application in tissue repair and regeneration, OE-MSCs have been used as a convenient and effective tool for the regeneration of hippocampal neuronal networks in mice and the spinal cord in humans.^16^ We previously reported that OE-MSCs can perform their immunosuppressive function by modulating T cell responses, while adoptive transfer of OE-MSCs can reduce disease severity in mice with collagen-induced arthritis.^19^ Moreover, we further showed that the immunosuppressive function of OE-MSCs can be modulated by the inflammatory microenvironment.^20^ However, it remains unclear whether OE-MSC-Exos have any therapeutic potential in suppressing autoimmune pathogenesis.

In this study, we found that OE-MSC-Exos significantly enhanced the suppressive function of MDSCs and markedly reduced disease severity in ESS mice. Mechanistically, we demonstrated that exosome-derived IL-6 increased the immunosuppressive capacity of MDSCs by activating the STAT3 pathway. Further investigation revealed that OE-MSC-Exos released S100A4 bound to TLR4 on MDSCs and triggered autocrine production of IL-6, which further enhanced the immunosuppressive function of MDSCs. Collectively, these data suggest that exosomes derived from OE-MSCs perform an immunoregulatory function and may be used as a novel strategy for the treatment of SS.

## Materials and methods

### Mice

Female C57BL/6 mice at 8 weeks of age were obtained from the Experimental Animal Center of Yangzhou University (Jiangsu, China) and maintained in the Animal Center of Jiangsu University (Jiangsu, China). All experiments were approved by the Committee on the Use of Live Animals in Research and Teaching of Jiangsu University.

### Isolation and culture of OE-MSCs and BM-MSCs

OE-MSCs were isolated from the nasal cavity of wild-type mice as we previously described.^19^ Briefly, cells were cultured in flasks with medium (Dulbecco’s modified Eagle’s medium (DMEM)/Ham’s F-12 supplemented with 15% fetal calf serum) (Gibco, Carlsbad, CA) and further expanded for three passages. For culture of bone marrow mesenchymal stem cells (BM-MSCs), bone marrow cells were collected by flushing the cells out of the femurs and tibiae of wild-type mice with cold phosphate-buffered saline (PBS) and culturing them in medium (DMEM supplemented with 15% fetal calf serum) (Gibco, Carlsbad, CA) for 3 days. Nonadherent cells were then removed, and when the remaining cells reached 80% confluence in the dish, the adherent cells were expanded for three passages and used for subsequent experiments. The purities of both OE-MSCs and BM-MSCs were greater than 95% according to the defined phenotype for MSCs with CD29, CD90, CD44, CD34, CD45, and CD11b expression^19,20^.

### Isolation of OE-MSC-Exos and BM-MSC-Exos

OE-MSCs and BM-MSCs were washed three times with PBS, moved to conditioned medium supplemented with exosome-depleted FBS produced by centrifugation at 120,000 × *g* for 18 h at 4 °C. The culture supernatants were collected after 48 h. Exosomes were purified from the supernatants by differential centrifugation at 300 × *g* for 10 min, 2000 × *g* for 10 min, and 10,000 × *g* for 30 min to remove residual cells and debris. Then, the supernatants from the final centrifugation were ultracentrifuged at 100,000 × *g* (Beckman Coulter, California, USA) for 1 h at 4 °C. After removing the supernatants, the exosomal pellets were washed in PBS and centrifuged at 100,000 × *g* for another 1 h at 4 °C, resuspended in PBS and stored at −80 °C. The size distribution of both types of MSC-Exos was measured by nanoparticle tracking analysis (NTA, Particle Metrix, Meerbusch, Germany); the protein content in both types of MSC-Exos was quantified using a micro-BCA protein assay kit; visualization of MSCs and exosomes was performed by transmission electron microscopy (TEM, Tecnai-12; Philips, Amsterdam, Netherlands) and scanning electron microscopy (SEM, XL-30ESEM, Philips, Amsterdam, Netherlands). The expression of the exosomal markers CD9 and CD63 and the negative marker calnexin (Abcam, Cambridge, MA, USA) was examined by Western blot analysis.

To extract exosomes from OE-MSCs with IL-6 or S100A4 knockdown, IL-6 or S100A4 siRNA (RiboBio Co, Guangzhou, China) was transfected into OE-MSCs, and exosomes were then extracted from the modified cells following the manufacturer’s protocol. The siRNA target sequences were as follows: IL-6 siRNA, GCTACCAAACTGGATATAA; S100A4 siRNA, TGAGCAACTTGGACAGCAA.

### Flow cytometric analysis

For evaluation of surface markers, single-cell suspensions were stained with relevant fluorochrome-conjugated monoclonal antibodies (mAbs): anti-mouse CD40 (clone HM40-3), CD80 (clone 16-10A1), CD86 (clone GL1), and MHCII (clone M5/114.15.2) from eBioscience (San Diego, CA, USA); anti-mouse CD11b (clone M1/70), Gr-1 (clone RB6-8C5), Ly6G (clone 1A8), and Ly6C (clone HK1.4) from Biolegend (San Diego, CA, USA). The oxidation-sensitive dye 29,79-dichlorofluorescein diacetate (Invitrogen, Carlsbad, CA, USA) was used to detect ROS production by MDSCs. For the detection of mouse Th1 cells, Th17 cells and expression of IL-6 in mouse MDSCs, cells were stimulated with PMA (Sigma-Aldrich, St. Louis, MO; 50 ng/mL), ionomycin (Enzo, Raamsdonksveer, The Netherlands; 1 µg/mL) and monensin (Enzo, 2 µg/mL) at 37 °C for 5 h. Single cells from mice were stained with an anti-CD4 mAb (clone RM4-5, eBioscience), fixed, permeabilized, and stained with an anti-IFN-γ mAb (clone XMG1.2, eBioscience) or an anti-IL-17 mAb (clone eBio17B7, eBioscience) according to the instructions of the Intracellular Staining Kit (Invitrogen); MDSCs were stained with an anti-mouse IL-6 antibody (clone MP5-20F3, eBioscience) using the same staining kit. Flow cytometry was performed using a BD FACSCanto II (Becton Dickinson, NJ, USA), and data were analyzed using FlowJo software (TreeStar, Ashland, OR).

### ESS induction and treatment with OE-MSC-Exos or BM-MSC-Exos

The ESS mouse model was induced as we previously described.^21^ Briefly, bilateral SGs were isolated from female C57BL/6 mice for homogenization in PBS to prepare SG proteins. Naive mice were immunized with 200 mg of SG proteins emulsified in Freund’s complete adjuvant (Sigma-Aldrich, St. Louis, MO) via subcutaneous injection in the neck on days 0 and 7. On day 14, a booster injection of SG proteins emulsified in incomplete Freund’s adjuvant (Sigma-Aldrich) was administered at a dose of 1 mg/ml. To determine the effects of OE-MSC-Exo treatment, mice were administered two intravenous injections of OE-MSC-Exos or BM-MSC-Exos (100 μg) on days 18 and 25 after the first immunization. The control group was administered the same volume of PBS at the same time points.

### Quantitative real-time PCR

Quantitative real-time PCR was performed as previously described.^9^ The sequences of the primers used were as follows: IL-6, forward 5′-CTGCAAGAGACTTCCATCCAG′-3′, reverse 5′-AGTGGTATAGACAGGTCTGTTGG′-3′; β-actin, forward 5′-TGGAATCCTGTGGCATCCATGAAAC-3′, reverse 5′-TAAAACGCAGCTCAGTAACAGTCCG-3′. β-Actin was used as the internal control.

### Preparation of MDSCs

CD11b^+^Gr-1^+^ MDSCs were isolated from the spleens of ESS mice using a BD FACSAria II SORP cell sorter. M-MDSCs and PMN-MDSCs were isolated using a Mouse MDSC Isolation Kit (Miltenyi Biotec, MA, Germany) following the manufacturer’s protocol.^9^

The purities of MDSCs, M-MDSCs, and PMN-MDSCs were greater than 90%.

### Autoantibody and cytokine detection

The levels of autoantibodies against SG proteins, the M3 muscarinic receptor (M3R), and Sjögren’s syndrome-related antigen A (SSA) were measured via sandwich enzyme-linked immunosorbent assay (ELISA), as previously described.^21^ Briefly, 96-well plates (Costar, St. Louis, USA) were coated with antigens (5 μg/mL in coating buffer) at 4 °C overnight. The plates were washed and blocked with blocking buffer at room temperature for 1 h. Samples were then added and incubated at room temperature for 2 h prior to incubation with biotin-conjugated anti-mouse IgG. After washing, HRP-streptavidin was added and incubated for 30 min. Then, the plates were washed, and TMB substrate was added. After 10 min, stop solution was added, and the absorbance at 450 nm was measured using a microplate reader (BioTek, Winooski, USA). Antigenic peptides of SSA (AVALREYRKKMDIPA) and M3R (VLVNTFCDSCIPK-TYWNLGY) were synthesized chemically by a solid phase approach and purified by high-performance liquid chromatography (SBS Genetec Co, Ltd, China). Mouse serum levels of IL-17, IFN-γ, IL-6, GM-CSF, TNF-α, and IL-1β were measured with ELISA kits (eBioscience) following the manufacturer’s protocol. Arginase activity and the NO concentration were measured as previously described.^9^ Arginase activity for the conversion of arginine to ornithine and urea was determined by a quantitative colorimetric approach employing a QuantiChrom arginase assay kit (BioAssay Systems, Northern California, USA). Arginase activity was calculated according to the manufacturer’s instructions. The concentration of NO was assessed by determining the concentration of nitrite accumulated in culture supernatants using the colorimetric Griess reaction (Promega, Madison, WI).

### Measurement of saliva flow rates

Saliva flow rates were measured as we previously described.^21^ In brief, mice were anesthetized by i.p. injection with pilocarpine (Sigma-Aldrich) at a dose of 5 mg/kg body weight, and a 20-μl pipette tip was used to collect saliva from the oral cavity for 15 min at room temperature.

### Histologic analysis

After mice were euthanized, submandibular glands were collected and immediately fixed with 4% paraformaldehyde. Paraformaldehyde-fixed tissues were embedded in paraffin. Serial 4-μm sections were cut and stained with hematoxylin and eosin for morphologic examination. A lymphocytic focus was defined as a group of >50 lymphocytes. The focus score (FS) was classified as follows^22^: FS = 0, no lymphocytic infiltration; FS = 1, <1 lymphocytic focus per 4 mm^2^ region (0<FS < 1); FS = 2, <2 lymphocytic foci per 4 mm^2^ region; FS = 3, two or more lymphocytic foci per 4 mm^2^ region.

### Induction of MDSC differentiation

For MDSC induction^23,24^, BM cells were collected by flushing the cells out of femurs and tibiae from wild-type mice with cold PBS. BM cells were cultured in the presence of recombinant murine IL-6 (10 ng/ml, R&D Systems, Minneapolis, USA) and GM-CSF (10 ng/ml, R&D) for 3 days.

### T cell suppression assay

Mouse CD4^+^ T cells were sorted from wild-type mice using CD4 microbeads (Miltenyi Biotec, Bergisch Gladbach, Germany). After coincubation with OE-MSC-Exos or BM-MSC-Exos for 2 days, MDSCs were harvested and cocultured with CD4^+^ T cells labeled with carboxyfluorescein succinimidyl ester (CFSE, 5 mM; Invitrogen at a ratio of 1:1 in 96-well plates (Costar, NY, USA)) in the presence of anti-CD3 (eBioscience, 1 μg/ml) and anti-CD28 mAbs (eBioscience, 1 μg/ml) for 3 days. The CFSE fluorescence intensity was analyzed to evaluate the proliferation of CD4^+^ T cells by flow cytometry.

### LC-MS/MS analysis

OE-MSC-Exos were lysed in STD buffer and centrifuged at 1000 × *g* for 10 min to collect the supernatants. Proteins were identified using a Q Exactive Orbitrap LC-MS/MS system (Thermo Fisher Scientific, MA, USA).

### Detection of exosome transfer into MDSCs

To detect the direct transfer of exosomes into MDSCs, both types of MSC-Exos were labeled using a PKH67 Fluorescent Cell Linker Kit (Sigma-Aldrich) and were then incubated with MDSCs for 6 or 12 h. Fluorescence signals were detected under an Olympus FluoView FV1000 confocal microscope.

### Western blot analysis

Protein extracted from cells was prepared as previously described.^9^ Equal amounts of protein were separated by 12% SDS-PAGE and transferred onto Immobilon polyvinylidene difluoride membranes (Bio-Rad, CA, USA). Antibodies against STAT3, p-STAT3, p-JAK2, JAK2, p-Akt, Akt, and S100A4 were purchased from Cell Signaling Technology (MA, USA).

### Statistical analysis

Statistical significance was evaluated by Student’s *t*-test or one-way ANOVA/two-way ANOVA. Correlations were assessed by computing the Spearman correlation coefficient. All analyses were performed using SPSS 16.0 software (IBM, NY, USA). *P* values of <0.05 were considered statistically significant.

## Results

### Isolation and identification of exosomes secreted from OE-MSCs and BM-MSCs

**Fig. 1:**
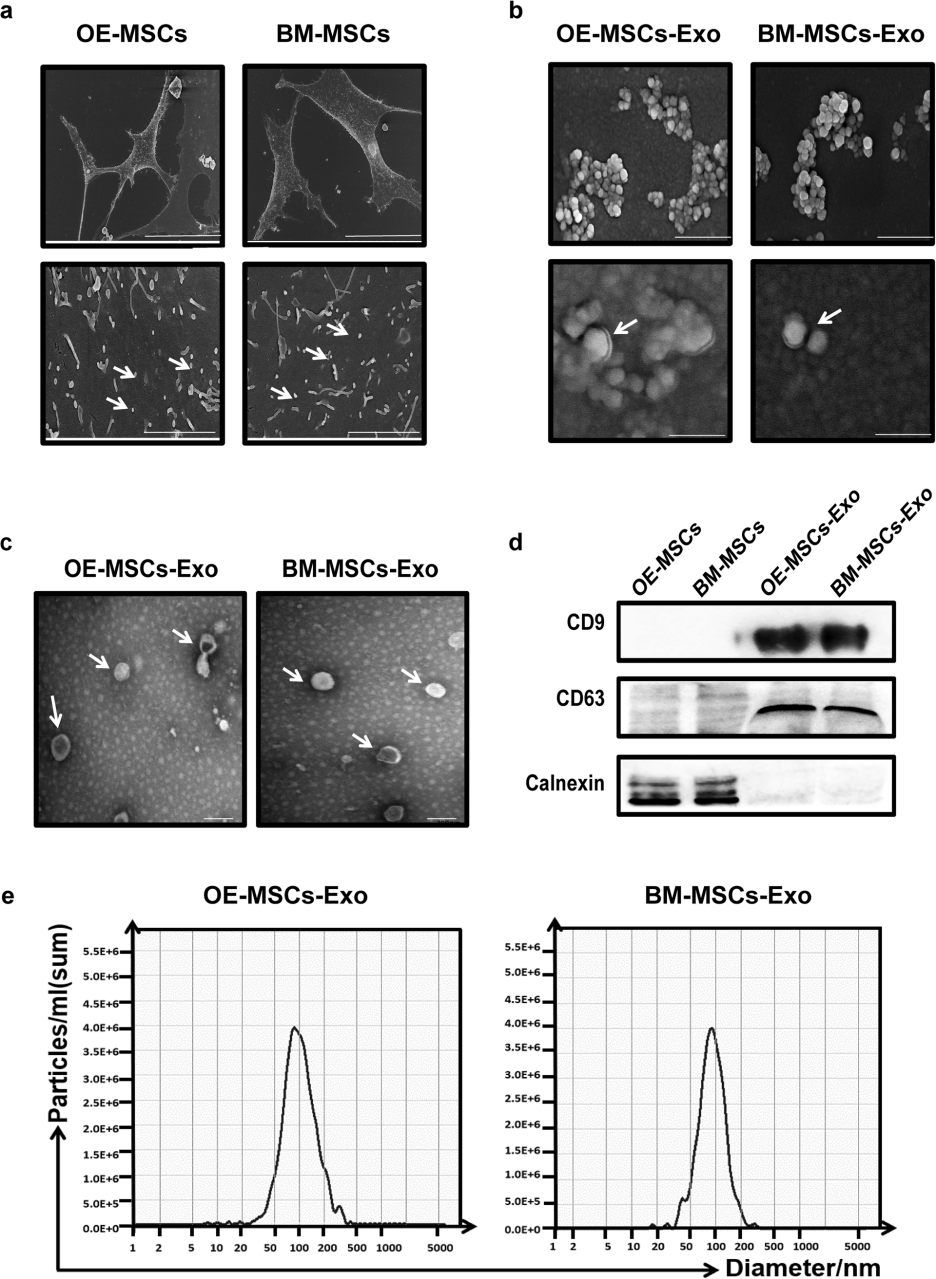
Isolation and identification of exosomes secreted by OE-MSCs and BM-MSCs. **a** OE-MSCs and BM-MSCs cultured under exosome culture conditions were visualized by SEM (upper bar = 50 μm, lower bar = 4 μm). Representative SEM (**b**) (upper bar = 500 nm, lower bar = 100 nm) and TEM (**c**) (bar = 200 nm) images of exosomes derived from OE-MSCs and BM-MSCs. **d** The expression of specific markers of OE-MSC-Exos and BM-MSC-Exos, including CD63, CD9, and calnexin (negative marker), was analyzed by western blotting. **e** The average sizes of OE-MSC-Exos and BM-MSC-Exos were determined by NTA

To characterize MSC-Exos, vesicle samples harvested from culture supernatants of OE-MSCs and BM-MSCs were morphologically assessed by SEM and TEM. As shown in Fig. 1a, vesicles harvested from OE-MSCs and BM-MSCs exhibited spherical double-membrane structures with a size of ~50–150 nm (Fig. 1b, c), the expected size of exosomes, as evaluated by particle size analysis with NTA (Fig. 1e). Moreover, Western blot analysis revealed that these exosomes expressed CD63 and CD9 but not calnexin, a typical phenotype of exosomes (Fig. 1d). Thus, these vesicles exhibited both morphologic and phenotypic characteristics of exosomes and could be isolated from the supernatants of cultured OE-MSCs and BM-MSCs.

### OE-MSC-Exos efficiently promote MDSC differentiation with enhanced suppressive function

**Fig. 2:**
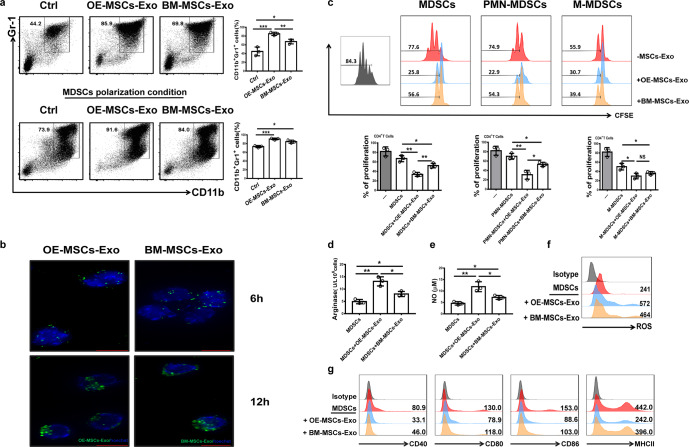
OE-MSC-Exos promote the expansion and immunosuppressive function of MDSCs in vitro. **a** BM cells were cocultured with OE-MSC-Exos or BM-MSC-Exos in the absence or presence of MDSC polarization medium (GM-CSF and IL-6) for 3 days. Flow cytometry was performed to analyze the expansion of CD11b^+^Gr-1^+^ MDSCs. **b** The localization of PKH67-labeled MSC-Exos (green) in MDSCs was observed 6 and 12 h after exosome treatment. Fluorescence signals were detected by fluorescence microscopy (bar = 10 μm). **c** MDSCs (PMN-MDSCs and M-MDSCs) isolated from the spleens of ESS mice 35 days after the first immunization with SG proteins were treated with OE-MSC-Exos or BM-MSC-Exos for 48 h, and MDSCs were then collected for coculture with CD4^+^ T cells in the presence of anti-CD3 and anti-CD28 mAbs for 72 h (MDSC:T cell ratio, 1:1). CD4^+^ T cell proliferation was evaluated by staining with CFSE. MSC-Exo-treated MDSCs were used to measure arginase activity (**d**), NO levels (**e**), and ROS levels (**f**). **g** The expression of CD40, CD80, CD86, and MHCII in both types of MSC-Exo-treated MDSCs was analyzed by flow cytometry. The concentration of both types of MSC-Exos was 60 μg/mL. The data are shown as the mean ± SD of three independent experiments. One-way ANOVA, ***p < 0.001, **p < 0.01, *p < 0.05. NS not significant

We examined whether OE-MSC-Exos can regulate the differentiation and function of MDSCs in culture. Mouse BM cells were cultured with MSC-Exos under MDSC polarization conditions. As shown in Fig. 2a, culture with OE-MSC-Exos and BM-MSC-Exos efficiently increased the frequencies of CD11b^+^Gr-1^+^ cells in the cultures. Notably, OE-MSC-Exos exhibited a much stronger capacity to promote the expansion of MDSCs in culture than did BM-MSC-Exos.

As early as 6 h after MSC-Exo treatment, the internalization of PKH67-labeled MSC-Exos in MDSCs was observed by fluorescence microscopy (Fig. 2b). After OE-MSC-Exo treatment, the suppressive effects of MDSCs on the proliferation of CD4^+^ T cells were markedly enhanced, while the activity of arginase and the levels of NO and ROS in MDSCs were significantly increased (Fig. 2c–f). Furthermore, OE-MSC-Exo treatment markedly reduced the expression levels of CD40, CD80, CD86, and MHCII in MDSCs (Fig. 2g). Taken together, these data indicated that OE-MSC-Exos can induce the differentiation and expansion of MDSCs with significantly enhanced immunosuppressive function.

We next investigated the effect of OE-MSC-Exo-treated MDSCs on inhibiting ESS progression in vivo. MDSCs treated with or without MSC-Exos were adoptively transferred into ESS mice on days 18 and 25 (Supplementary Fig. 1a). Compared with mice in the control MDSC group, ESS mice exhibited significantly increased saliva flow rates and decreased levels of autoantibodies after transfer of either type of MSC-Exo-treated MDSCs (Supplementary Fig. 1b–e). In addition, mice in the OE-MSC-Exo-MDSC group showed less lymphocyte infiltration in the submandibular glands (Supplementary Fig. 2f, g), with markedly decreased Th1 and Th17 responses (Supplementary Fig. 2h). Taken together, these findings indicate that OE-MSC-Exos can enhance the immunosuppressive function of MDSCs and inhibit disease progression in ESS mice.

### Adoptive transfer of OE-MSC-Exos inhibits disease progression and Th1/Th17 responses in ESS mice

**Fig. 3:**
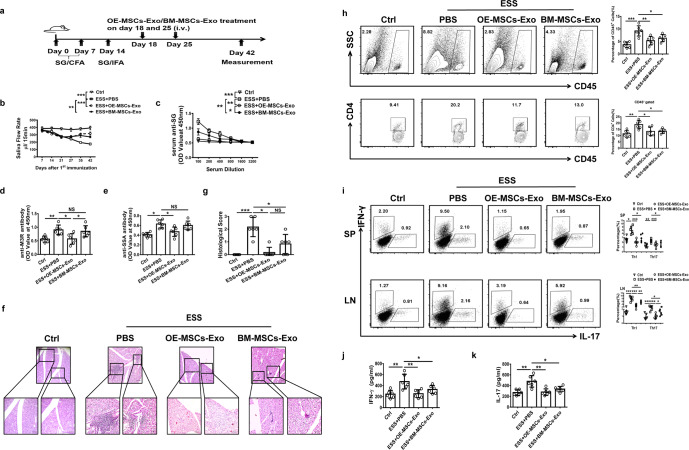
Adoptive transfer of OE-MSC-Exos alleviates the progression of ESS by suppressing Th1/Th17 responses. **a** Graphical schematic of ESS induction and OE-MSC-Exo/BM-MSC-Exo administration. ESS mice were immunized with SG proteins, and mice in the treatment groups were intravenously injected with OE-MSC-Exos or BM-MSC-Exos on days 18 and 25 after the first immunization. **b** The saliva flow rates were measured in each group (*n* = 6). Autoantibodies against SG antigens (**c**), M3R (**d**), and SSA (**e**) in the serum of mice (*n* = 6) were analyzed. **f** Histological evaluation of glandular destruction in each group was performed on tissue sections of submandibular glands stained with H&E 10 weeks after the first immunization (upper bar = 150 μm, lower bar = 50 μm). **g** Histological scores were assessed based on lymphocytic infiltration in the SGs. **h** Representative flow cytometric profiles of infiltrating CD45^+^ leukocytes and CD4 T cells in the SGs in different treatment groups on day 10. **i** The proportions of CD4^+^IFN-γ^+^ Th1 cells and CD4^+^IL-17^+^ Th17 cells in the spleen (SP) and cervical lymph nodes (CLN) of mice in different treatment groups on day 42 (*n* = 6) were determined. Serum levels of IFN-γ (**j**) and IL-17 (**k**) were measured in the different groups on day 42 (*n* = 6). The data are shown as the mean ± SD of three independent experiments. Two-way ANOVA was performed in **b** and **c**; one-way ANOVA in **d**, **e**, **g**–**k**. ***p < 0.001, **p < 0.01, *p < 0.05. NS not significant

To evaluate the therapeutic potential of MSC-Exos in ESS, we adoptively transferred both types of MSC-Exos into ESS mice and compared their therapeutic effects (Fig. 3a). Remarkably, the reduced saliva flow rates in ESS mice were significantly improved following treatment with either type of MSC-Exos (Fig. 3b). Moreover, the serum levels of autoantibodies against total SG antigens, SSA, and M3R were markedly decreased in the OE-MSC-Exo-treated group compared with the BM-MSC-Exo-treated group and control group (Fig. 3c–e). Histopathological assessment showed reduced pathological changes in the submandibular glands of ESS mice treated with either BM- or OE-MSC-Exos. As shown in Fig. 3g, f, only a small amount of lymphocytic infiltration was observed in SGs after treatment with either type of MSC-Exos. Moreover, OE-MSC-Exo-treated mice showed significantly decreased histological scores of SG destruction compared with those of BM-MSC-Exo-treated or untreated ESS mice. Consistent with the histological observations, we found a significantly lower proportion of CD45^+^ leukocytes and CD4^+^ T cells in the SG of OE-MSC-Exo-treated ESS mice (Fig. 3h). In addition, in ESS mice with OE-MSC-Exo treatment, the frequencies of Th1 and Th17 cells were significantly reduced compared with those in untreated ESS mice (Fig. 3i). Consistent with this result, the serum levels of the inflammatory cytokines IFN-γ and IL-17 were decreased in OE-MSC-Exo-treated ESS mice (Fig. 3j, k). Collectively, these data demonstrated that OE-MSC-Exo treatment exerted an immunosuppressive effect and attenuated disease progression in ESS mice.

### Adoptive transfer of OE-MSC-Exos promotes the suppressive function of MDSCs in ESS mice

**Fig. 4:**
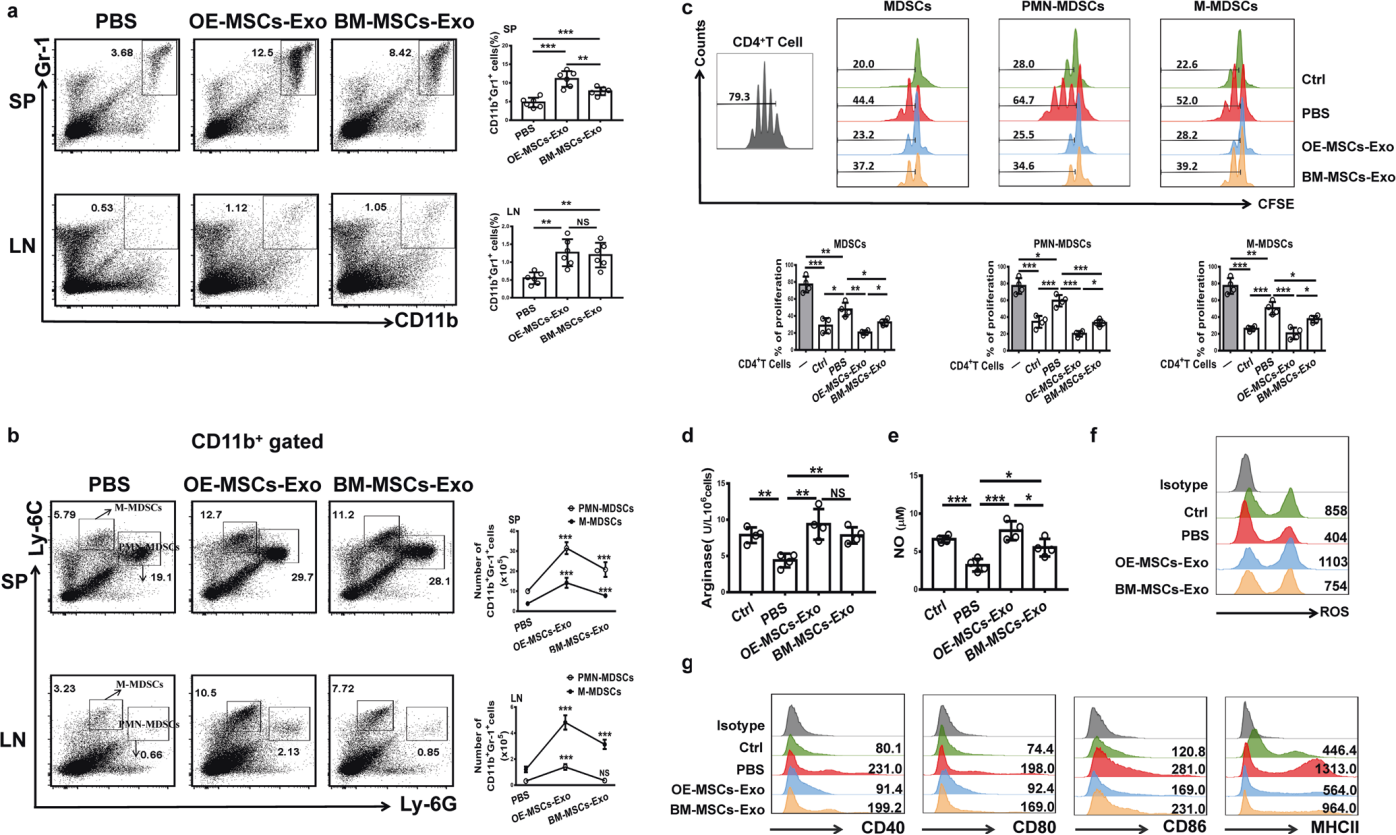
Treatment with OE-MSC-Exos increases the percentages and function of MDSCs in ESS mice. **a** The percentages of CD11b^+^Gr-1^+^ MDSCs were determined in the SP and CLNs after OE-MSC-Exo or BM-MSC-Exo treatment (*n* = 6). **b** The proportions and numbers of M-MDSC and PMN-MDSC subsets in the SP and CLNs were evaluated in the groups treated with both types of MSC-Exos (*n* = 6). **c** MDSCs from the groups treated with both types of MSC-Exos were collected for coculture with CD4^+^ T cells in the presence of anti-CD3 and anti-CD28 mAbs for 72 h (MDSC:T cell ratio, 1:1). CD4^+^ T cell proliferation was evaluated by staining with CFSE (*n* = 4). Control MDSCs were prepared from mice 5 days after the first immunization. The activity of arginase (**d**) and the levels of NO (**e**) and ROS (**f**) were evaluated in different groups (*n* = 4). **g** The levels of CD40, CD80, CD86, and MHCII were measured in MDSCs isolated from the groups treated with both types of MSC-Exos. The data are shown as the mean ± SD of three independent experiments; one-way ANOVA. ***p < 0.001, **p < 0.01, *p < 0.05. NS not significant

We next assessed the frequencies and suppressive function of MDSCs in ESS mice after MSC-Exo treatment. As shown in Fig. 4a, we found a marked expansion of CD11b^+^Gr-1^+^ MDSCs in the MSC-Exo-treated groups. Moreover, both the numbers and proportions of PMN-MDSCs and M-MDSCs were significantly increased in the MSC-Exo-treated groups (Fig. 4b). Notably, OE-MSC-Exo treatment enhanced the suppressive effect of MDSCs on CD4^+^ T cells more potently than did BM-MSC-Exo treatment (Fig. 4c) and resulted in higher levels of arginase expression and NO and ROS production (Fig. 4d–f). Interestingly, compared to BM-MSC-Exo treatment OE-MSC-Exo treatment modulated MDSCs with an immature phenotype showing low levels of CD40, CD80, CD86, and MHCII expression (Fig. 4g). Taken together, these results indicate that OE-MSC-Exo treatment promotes MDSC expansion and enhances the suppressive effects of MDSCs in ESS mice.

### Exosome-secreted IL-6 enhances the immunosuppressive function of MDSCs

**Fig. 5:**
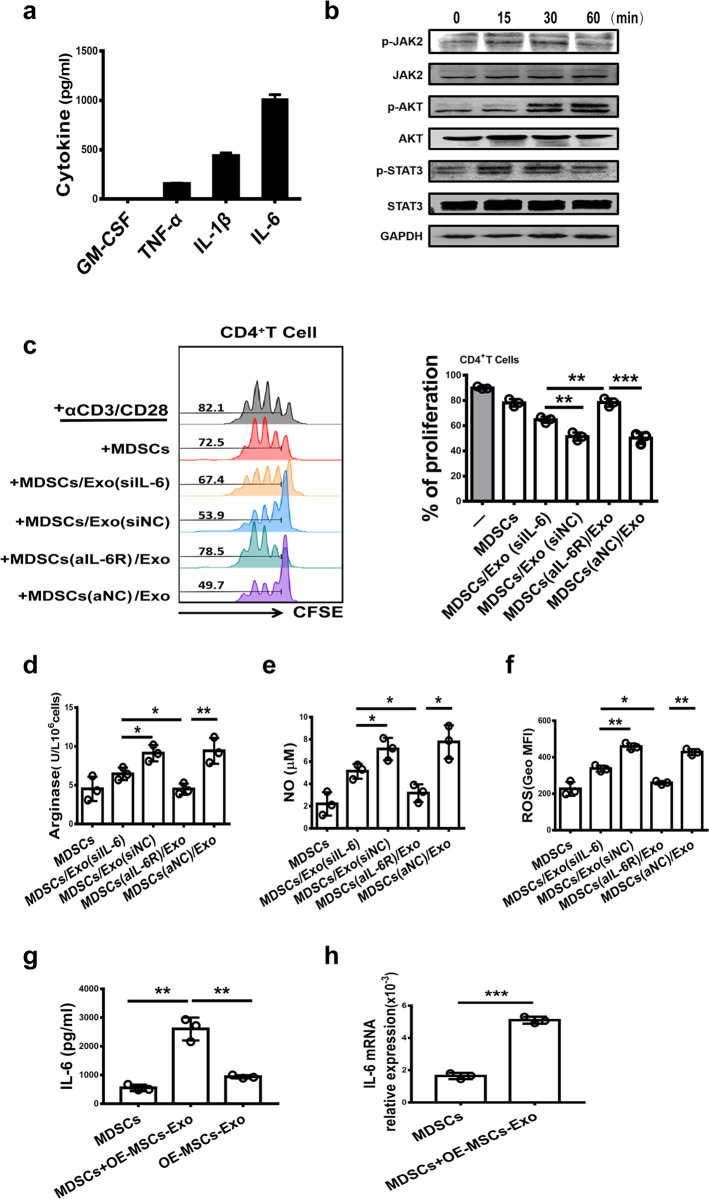
The immunosuppressive function of MDSCs can be enhanced by exosomes secreting IL-6 and by the IL-6 autocrine signaling pathway. **a** Detection of various cytokines (GM-CSF, TNF-α, IL-1β, and IL-6) in OE-MSC-Exos by ELISA. **b** The levels of phosphorylated STAT3, JAK2, and AKT in OE-MSC-Exo-treated MDSCs were determined by western blot analysis. **c** MDSCs were treated with OE-MSC-Exos without IL-6 or were pretreated with an anti-IL-6R antibody prior to OE-MSC-Exos treatment, and MDSCs from different groups were collected for coculture with CD4^+^ T cells in the presence of anti-CD3 and anti-CD28 mAbs for 72 h (MDSC:T cell ratio, 1:1). CD4^+^ T cell proliferation was evaluated by staining with CFSE. The expression of arginase (**d**) and the levels of NO (**e**) and ROS (**f**) in MDSCs were measured. **g**, **h** The levels of IL-6 secreted by MDSCs, secreted by OE-MSCs-Exos and in the supernatant of MDSCs treated with OE-MSC-Exos were measured by ELISA (**g**). qRT-PCR was used to analyze IL-6 mRNA expression in MDSCs treated with or without OE-MSC-Exos (**h**). The concentration of OE-MSC-Exos was 60 μg/mL. The data are shown as the mean ± SD of three independent experiments. One-way ANOVA in **c**–**g**; Student’s *t*-test in **h**. ***p < 0.001, **p < 0.01, *p < 0.05. NS not significant

To further elucidate the mechanism underlying the OE-MSC-Exo-mediated effects on MDSCs, we analyzed exosome-derived effector molecules involved in modulating the suppressive function of MDSCs and found a high level of IL-6 in OE-MSC-Exos (Fig. 5a). Moreover, the IL-6 signaling-mediated Jak2/Stat3 pathway was rapidly activated in MDSCs after OE-MSC-Exo treatment (Fig. 5b). To further examine the role of exosome-derived IL-6 in regulating the suppressive function of MDSCs, we blocked the IL-6/IL-6R pathway by knocking down IL-6 in OE-MSC-Exos with siRNA or using an anti-IL-6R antibody when coculturing MSC-Exos with MDSCs. Interestingly, both interventions for IL-6 blockade markedly abolished the enhanced immunosuppressive effects of OE-MSC-Exos on MDSCs, but anti-IL-6R treatment exhibited a more significant effect than siRNA treatment on MDSCs. As shown in Fig. 5c, the suppressive effect of MDSCs on CD4^+^ T cells was partially decreased in the siIL-6 Exo group, whereas the suppressive function of MDSCs was completely abolished in the anti-IL-6R Ab group, with decreased arginase activity and NO and ROS levels (Fig. 5d–f). Furthermore, the levels of IL-6 in the culture supernatant of OE-MSC-Exo-treated MDSCs were significantly higher than those in the culture supernatant of control MDSCs or in OE-MSC-Exos, and IL-6 mRNA expression in MDSCs was strikingly increased after OE-MSC-Exo treatment (Fig. 5g, h). These results suggest that MDSCs can secrete more IL-6 after OE-MSC-Exo stimulation. Moreover, the secreted IL-6 can promote the function of MDSCs in an autocrine manner. Thus, the immunosuppressive function of MDSCs can be enhanced by both exosome-derived and MDSC-secreted IL-6 via an autocrine signaling pathway.

### OE-MSC-Exos induce IL-6 production in MDSCs via Toll-like receptor 4 (TLR4)

**Fig. 6:**
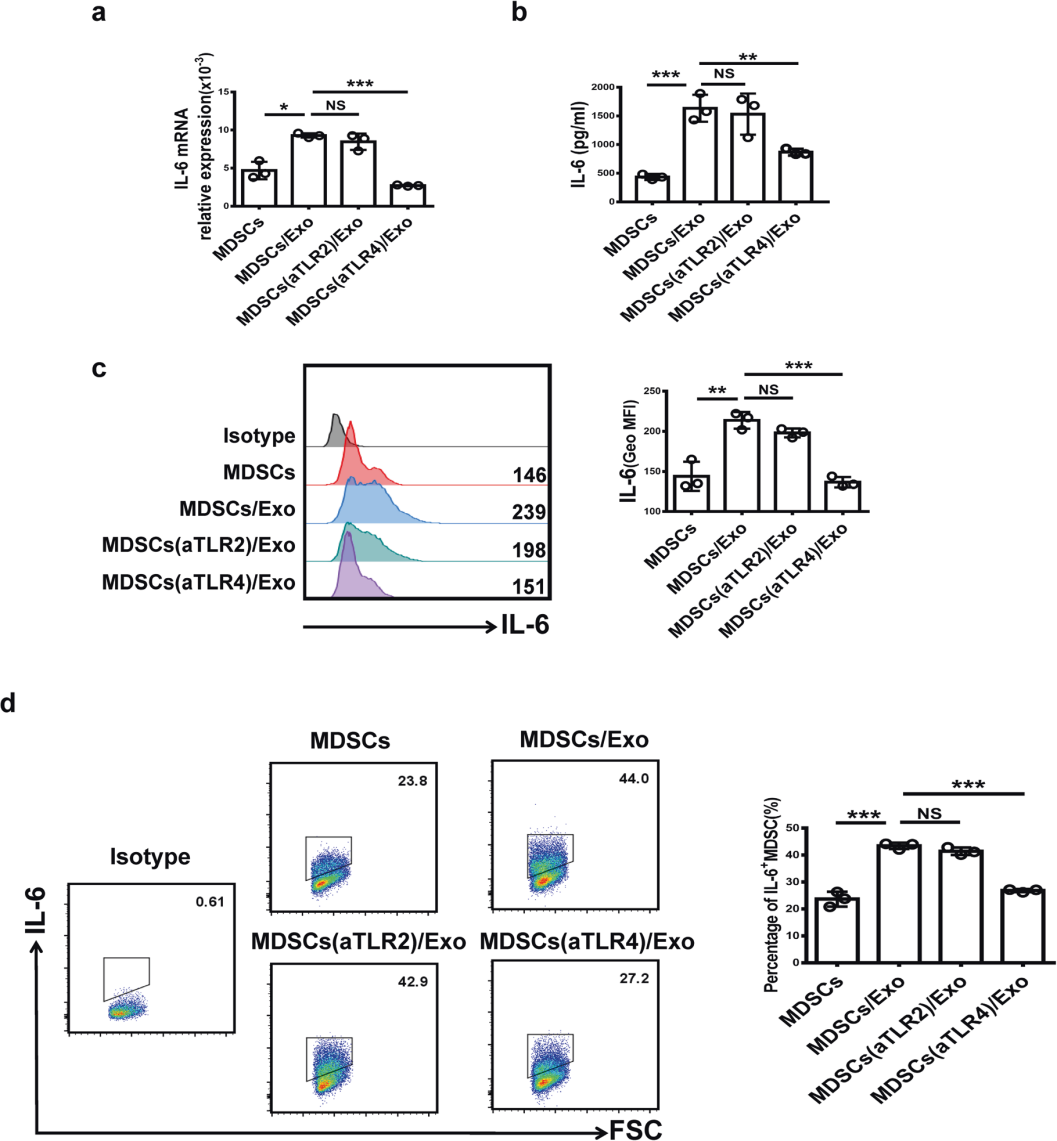
OE-MSC-Exo-induced IL-6 production in MDSCs is mediated by TLR4. MDSCs were pretreated with TLR2 or TLR4 neutralizing antibodies (2 μg/ml) for 2 h and were then treated with OE-MSC-Exos. The mRNA levels of IL-6 in MDSCs after different treatments were determined by qRT-PCR (**a**), and the levels of IL-6 secreted by MDSCs were measured by ELISA (**b**). **c** The expression of IL-6 in MDSCs was analyzed by flow cytometry. **d** The proportions of IL-6^+^ MDSCs after different treatments were determined by flow cytometry. The concentration of OE-MSC-Exos was 60 μg/mL. The data are shown as the mean ± SD of three independent experiments; one-way ANOVA. ***p < 0.001, **p < 0.01, *p < 0.05. NS not significant

Previous studies have suggested that Toll-like receptors, including TLR2, 3, 4, 7, 8, and 9, are the main inducers of IL-6 production in myeloid cells.^25–27^ As shown in Supplementary Fig. 2, the levels of TLR2 and TLR4 transcripts were high, while those of TLR7, TLR8, and TLR9 were very low, and those of TLR3 were almost undetectable in MDSCs. Therefore, we focused on investigating the roles of TLR2 and TLR4 in this study. We then used TLR2 or TLR4 neutralizing antibodies to analyze the functions of TLR2 and TLR4 in the induction of IL-6 expression in MDSCs. As shown in Fig. 6a–c, blocking TLR4 but not TLR2 significantly reduced IL-6 expression at both the mRNA and protein levels in MDSCs. After TLR4 pathway inhibition, the percentage of IL-6^+^ MDSCs was significantly decreased (Fig. 6d).

### MSC-Exo-secreted S100A4 induces IL-6 production in MDSCs

**Fig. 7:**
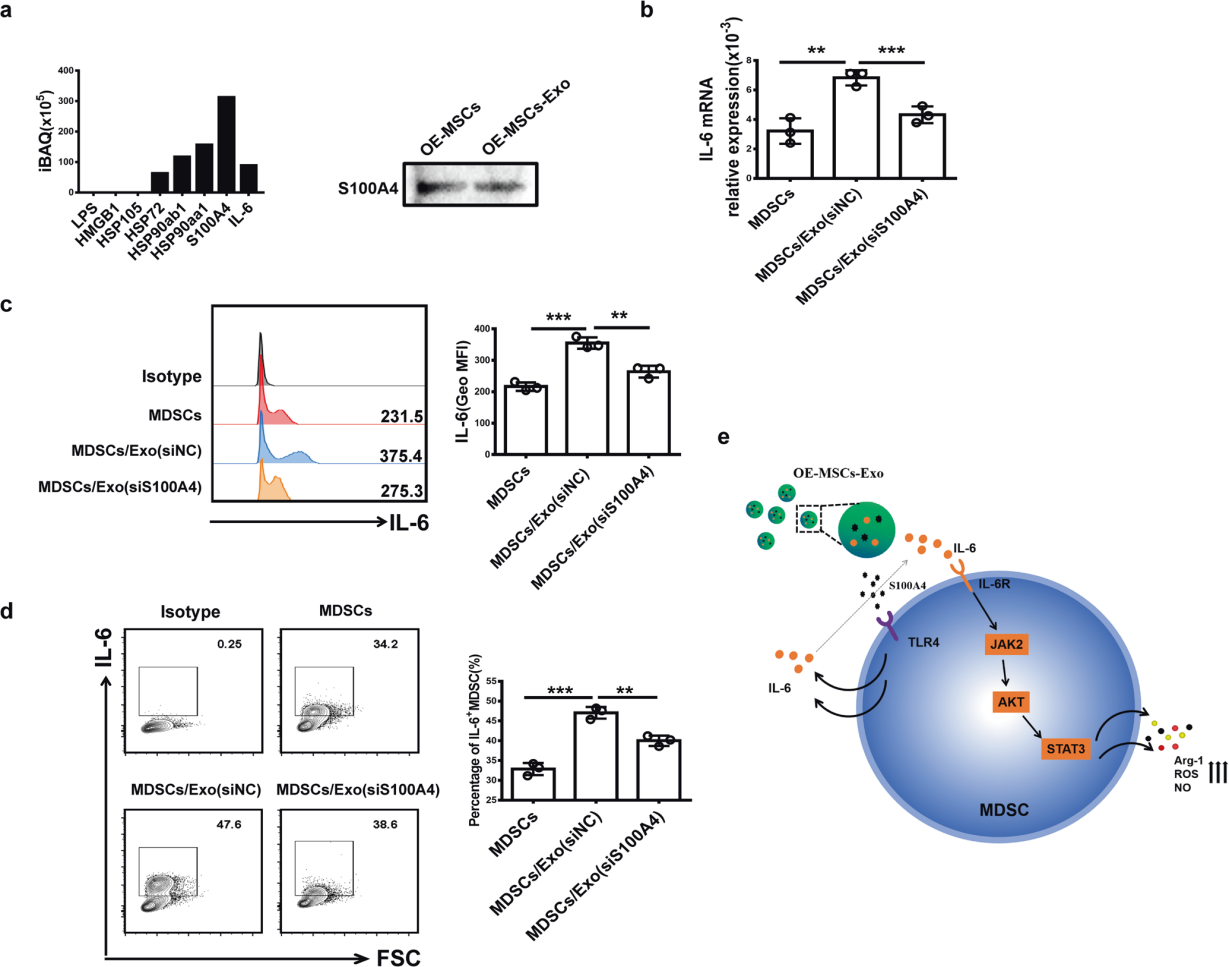
OE-MSCs-Exo-derived S100A4 induces IL-6 production in MDSCs. **a** LC-MS/MS proteomic analyses of OE-MSC-Exos were performed, and the results were confirmed by western blotting. **b**, **c** qRT-PCR and western blotting were used to analyze the mRNA and protein levels of IL-6 in MDSCs treated with OE-MSC-Exos with or without S100A4. **d** The proportions of IL-6^+^ MDSCs in different groups were measured by flow cytometry. **e** Proposed model describing the role of OE-MSC-Exos in regulating the immunosuppressive function of MDSCs. The concentration of OE-MSC-Exos was 60 μg/mL. The data are shown as the mean ± SD of three independent experiments; one-way ANOVA. ***p < 0.001, **p < 0.01, *p < 0.05. NS not significant

To further identify the potential ligands for TLR4 in OE-MSC-Exos, we performed proteomic analysis of OE-MSC-Exos and detected the abundant expression of S100A4, a ligand for TLR4, in OE-MSC-Exos (Fig. 7a). In addition, IL-6 was detected by proteomic analysis. To further confirm the role of S100A4 in promoting IL-6 production in MDSCs, we cultured MDSCs with exosomes prepared from S100A4-silenced OE-MSCs. Notably, the expression of S100A4 in exosomes was successfully knocked down (Supplementary Fig. 3). Moreover, IL-6 in MDSCs was markedly downregulated (Fig. 7b–d), suggesting that high levels of S100A4 from OE-MSC-Exos may contribute to the enhanced immunosuppressive function of MDSCs by inducing IL-6 production through TLR4 signaling.

## Discussion

In this study, we first showed that OE-MSC-Exos potently enhanced the immunosuppressive capacity of MDSCs in culture. Moreover, intravenous injection of OE-MSC-Exos significantly improved the saliva flow rate and reduced tissue damage in the SGs of ESS mice, in which OE-MSC-Exo treatment promoted MDSC expansion with enhanced immunosuppressive function. Further studies revealed that exosome-secreted IL-6 was critically involved in promoting the immunosuppressive function of MDSCs in an autocrine fashion. Collectively, these results demonstrated the therapeutic potential of OE-MSC-Exos via the modulation of MDSC function and suppression of autoimmune progression in ESS mice.

Exosomes are membrane microvesicles with a diameter of 40–100 nm and are secreted by most cell types; in addition, they have been shown to mediate cell-to-cell communication and participate in many processes, including inflammation, cell proliferation, differentiation and immune signaling.^14^ Exosomes can also act directly on the target cell membrane by fusion, transferring components into intracellular compartments or inducing endocytosis.^15^ Recent evidence supporting the exosomal paracrine hypothesis has introduced a different dimension for therapeutic applications of MSCs in regenerative medicine. Compared to MSCs, MSCs-Exos appear much safer for clinical application due to their superior stability with relatively low costs of storage, transport, and recovery. For instance, the application of MSC-Exos may mitigate the safety risks involving the replication of cells that may persist or be amplified over time after the cells are no longer needed or of uncontrolled cell growth after treatment has been terminated.^12,15^ In this study, we characterized OE-MSC-Exos as a novel mediator for modulating the function of MDSCs. Compared with the therapeutic effects exerted by BM-MSC-Exos, the effects of treatment with OE-MSC-Exos on improving the saliva flow rate and reducing SG damage in ESS mice were greatly enhanced. Remarkably, OE-MSC-Exo treatment significantly modulated the function of MDSCs with an immature phenotype and suppressed inflammatory Th1 and Th17 cell responses, reducing disease severity in ESS mice. Since the reductions in the percentages of Th1 and Th17 cells after OE-MSC-Exo treatment may be directly mediated by administered exosomes and indirectly regulated by MDSCs, further studies are needed to determine whether OE-MSC-Exos can directly suppress Th1 and Th17 cell responses in ESS mice. To date, clinical trials involving MSC-Exos have been conducted. In preclinical studies, MSC-Exos were shown to be safe and scalable to clinically relevant doses. With the rapid progress in bioengineering and cellular modification techniques, MSC-Exos with immunomodulatory functions may possess the potential for targeted clinical application in autoimmune diseases, including SS.

Recent studies have highlighted the critical involvement of MDSCs as a prominent leukocyte subpopulation not only in tumor-associated immune suppression but also in various autoimmune diseases.^8,28–32^ We previously showed that MDSCs exhibited gradually diminished suppressive capacity during the progression of ESS, while certain subsets of MDSCs even promoted an inflammatory response and tissue damage^9^, indicating a critical involvement of MDSCs in the development of ESS. These findings prompted us to explore whether OE-MSC-Exos can modulate the function of MDSCs and attenuate disease progression in ESS mice. Here, we found that OE-MSC-Exos efficiently promoted the expansion of MDSCs and enhanced the suppressive effect of MDSCs on T cell proliferation in vitro. In addition, adoptive transfer of OE-MSC-Exo-treated MDSCs led to reduced Th1 and Th17 cell responses, further suggesting the suppressive effect of OE-MSC-Exos on disease progression. Remarkably, OE-MSC-Exos modulated the function of MDSCs with an immature phenotype and increased the expression of inhibitory factors both in vivo and in vitro. Since recent studies have demonstrated the involvement of regulatory cells, such as Tregs and Bregs^33,34^, in the pathogenesis of SS, it remains to be investigated whether the functions of these regulatory cells can be modulated by the administered exosomes.

Extensive studies have identified IL-6 as a proinflammatory cytokine under pathological conditions, but other lines of evidence have suggested that IL-6 plays a multifaceted role during disease progression.^35–38^ The multiple effects of IL-6 in different cell types further illustrate the complexity of cytokine functions. It has been reported that IL-6 exerts a protective effect on macrophages to limit excessive inflammation in type 2 diabetes.^39^ Moreover, IL-6 was found to be a key effector in promoting the suppressive capacity of MDSCs.^40,41^ In this study, exosomes prepared from MSCs with IL-6 gene silencing showed markedly decreased effects on enhancing the immunosuppressive function of MDSCs, indicating that IL-6 released by OE-MSC-Exos is involved in enhancing the immunosuppressive function of MDSCs. Interestingly, significantly increased IL-6 production was detected in MDSCs upon OE-MSC-Exo treatment (Fig. 6). When IL-6R expressed on MDSCs was blocked by the neutralizing antibody, the effect of OE-MSC-Exos on enhancing the suppressive capacity of MDSCs was totally inhibited, suggesting that the IL-6 acting on MDSCs was not only derived from OE-MSC-Exos but also resulted from endogenous production by MDSCs upon OE-MSC-Exo treatment. Taken together, our results demonstrate a critical role of IL-6 in MSC-Exo-mediated enhancement of the immunosuppressive function of MDSCs.

It has been reported that TLR2 and TLR4 agonists can directly induce the production of IL-6 and other cytokines in myeloid cells.^25^ Shen et al. demonstrated that tumor-derived exosomes induce DCs to produce IL-6 in a TLR2- and TLR4-dependent manner.^42^ In addition, tumor-derived exosomes have been reported to trigger Stat3 activation in MDSCs to induce IL-6 secretion via the TLR2/MyD88 pathway.^40^ Here, we found significantly decreased IL-6 production in MDSCs upon blocking the TLR4 pathway, indicating that the endogenous production of IL-6 by MDSCs is mainly dependent upon the TLR4 pathway.

To identify the OE-MSC-Exo-derived ligand(s) involved in activating TLR4 on MDSCs, we examined the expression profile of exosomal proteins, including S100A4, a member of the S100 calcium-binding protein family.^43^ Previous studies have reported that extracellular S100A4 protects MDSCs from intrinsic apoptosis via TLR4-mediated ERK1/2 signaling.^44^ In addition, Cerezo et al. demonstrated that the S100A4 protein induced the production of IL-6 in mononuclear cells through TLR4 by activating the MyD88, NF-κB, and ERK1/2 pathways.^45^ Via proteomic analysis, we found that OE-MSC-Exos contained a high level of S100A4. Moreover, the secretion of IL-6 by MDSCs was inhibited after treatment with exosomes from MSCs with S100A4 gene silencing. Thus, these results suggest that Exo-derived S100A4 may act as a critical effector molecule in regulating the function of MDSCs.

In conclusion, our findings show that OE-MSC-secreted exosomes can potently promote the expansion of MDSCs with strong immunosuppressive function and effectively suppress ESS development by secreting IL-6 to enhance the function of MDSCs. In addition, OE-MSC-Exo-derived S100A4 promotes autocrine production of IL-6 in MDSCs to maintain their immunoregulatory functions. Taken together, our findings demonstrate the therapeutic potential of OE-MSC-Exos in SS and other autoimmune diseases.

## Supplementary information

Supplementary file

Supplementary Figure 1

Supplementary Figure 2

Supplementary Figure 3
