# Supplementary material for: Olfactory ecto-mesenchymal stem cell-derived exosomes ameliorate murine Sjögren’s syndrome by modulating the function of myeloid-derived suppressor cells
Source: Cell Mol Immunol. 2021 Jan 6;18(2):440–51. doi: 10.1038/s41423-020-00587-3 (PMC8027615; doi:10.1038/s41423-020-00587-3)
Supplement: Supplementary file 1 — Supplementary file [file 41423_2020_587_MOESM1_ESM.doc]

Olfactory ecto-mesenchymal stem cell-derived exosomes ameliorate murine Sjögren’s syndrome via modulating the function of myeloid-derived suppressor cells

**Supplementary Figures**

**
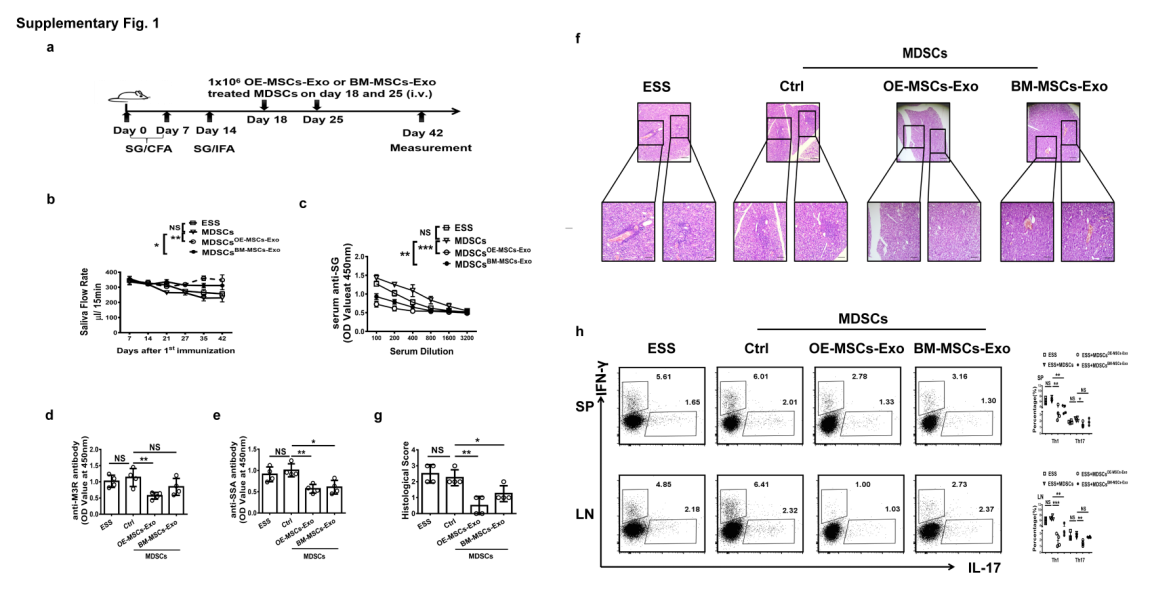
**

**Supplementary Figure 1. MDSCs treated with OE-MSCs-Exo recover their capacity ininhibiting ESS development.** **(a)** Graphic scheme of ESS induction and MDSC administration. MDSCs isolated from spleens of ESS mice on day 42 after the first immunization were treated with or without OE-MSCs-Exo or BM-MSCs-Exo (60μg/mL) for 48h, and 0.2ng/mL GM-CSF was added to sustain the survival of MDSCs. 1×10^6^ OE-MSCs-Exo or BM-MSCs-Exo treated MDSCs or Ctrl-MDSCs were intravenously injected on days 18 and 25 after the first immunization. Mice were sacrificed on day 42 (n=6/group). **(b)** The saliva flow rates were measured in each group. **(c-e)** Autoantibodies against SG antigens **(c)**, SSA **(d)** and anti-M3R antibodies **(e)** were analyzed in the serum of mice with different treatment. **(f)** The histological evaluation of glandular destruction in each group was performed on tissue sections of submandibular glands with H&E staining 10 weeks post first immunization. **(g)** Histological scores were assessed based on lymphocytic infiltration in the SG. **(h)** The proportions of CD4^+^IFN-γ^+^ Th1 cells and CD4^+^IL-17^+^ Th17 cells were measured in SP and CLN of mice with different treatment on day 42. The concentration of both MSCs-Exo was 60μg/mL.Data are shown as mean± SD of three independent experiments. Two-way ANOVA in b and c, one-way ANOVA in d, e, g and h ,***p < 0.001, **p < 0.01, *p < 0.05. N.S, no significance.


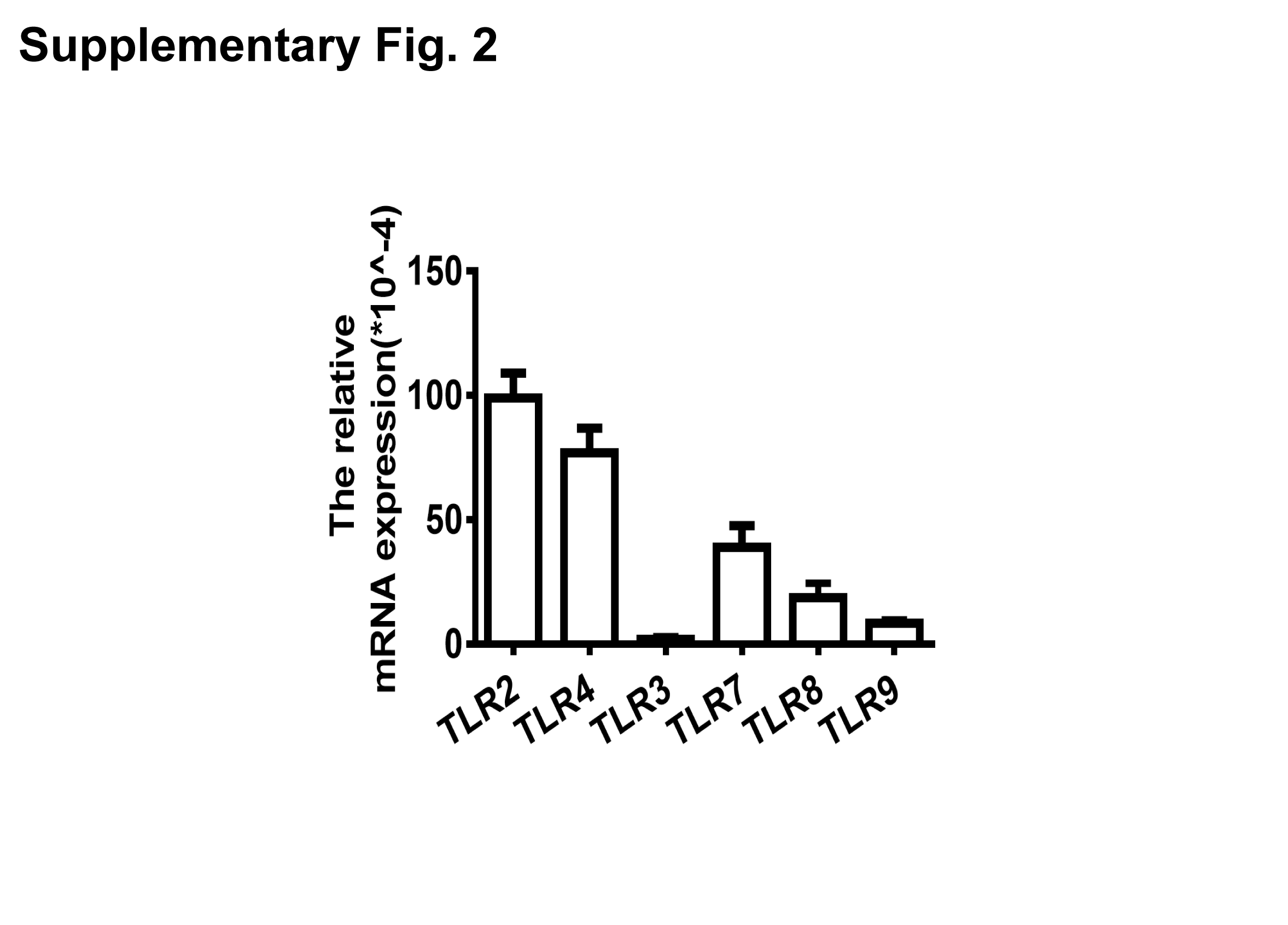


**Supplementary Figure 2. The mRNA levels of TLRs in MDSCs from ESS mice.** MDSCs isolated from ESS mice were subjected to analyze the mRNA expression of TLRs 2, 3, 4, 7, 8, 9 by qRT-PCR.


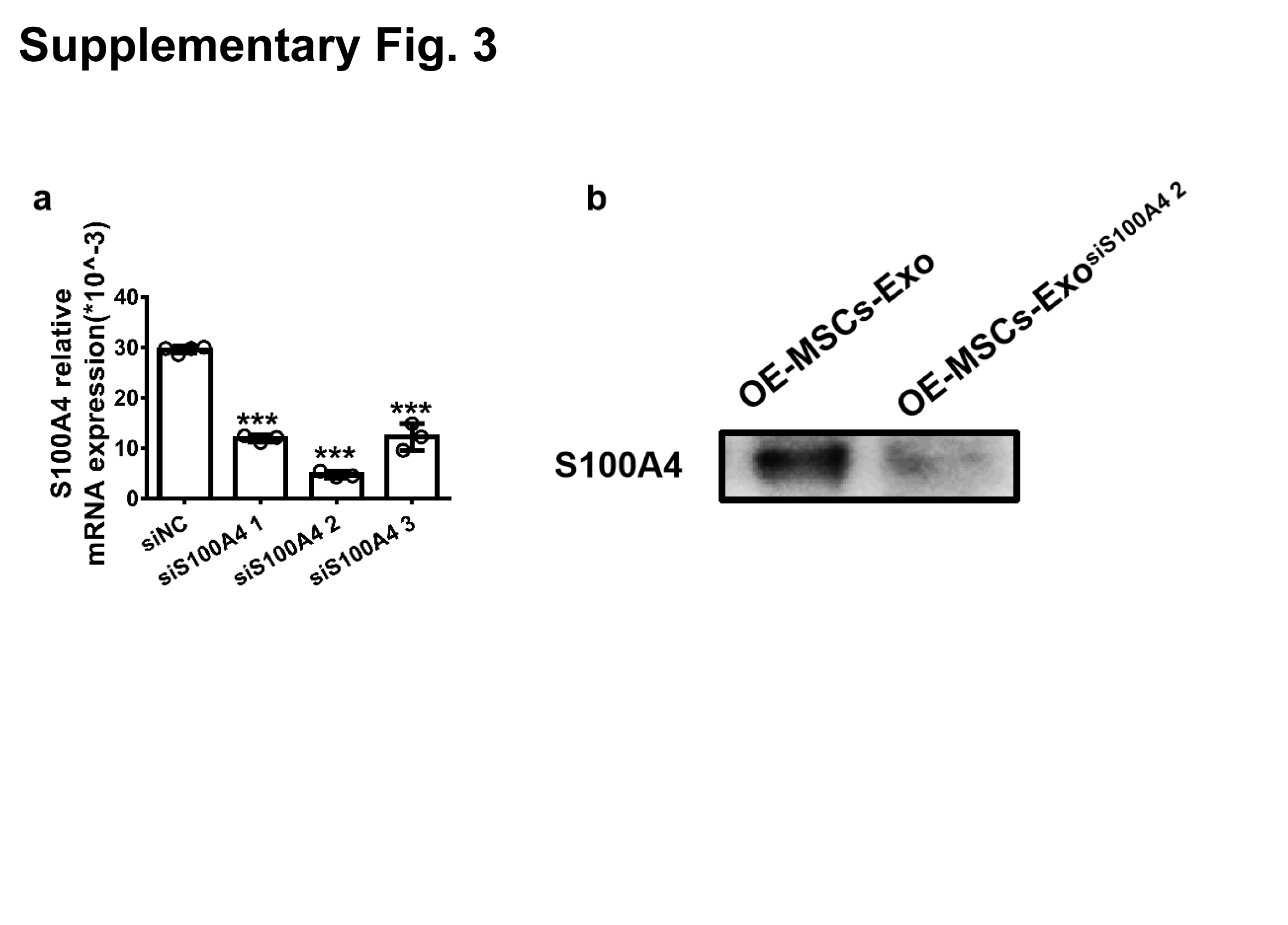


**Supplementary Figure 3. The interference efficiency of S100a4 in OE-MSCs-Exo. (a)** The mRNA levels of S100A4 in OE-MSCs interfered with siRNA or negative control were analyzed by qRT-PCR. **(b)** Western blot was used to analyze the level of S100A4 in exosomes prepared from S100A4-silenced OE-MSCs. Data are shown as mean±SD from three independent experiments with one-way ANOVA analysis, ***p < 0.001.
