# Supplementary figures and images for: Olfactory ecto-mesenchymal stem cell-derived exosomes ameliorate murine Sjögren’s syndrome by modulating the function of myeloid-derived suppressor cells
Source: Cell Mol Immunol. 2021 Jan 6;18(2):440–51. doi: 10.1038/s41423-020-00587-3 (PMC8027615; doi:10.1038/s41423-020-00587-3)

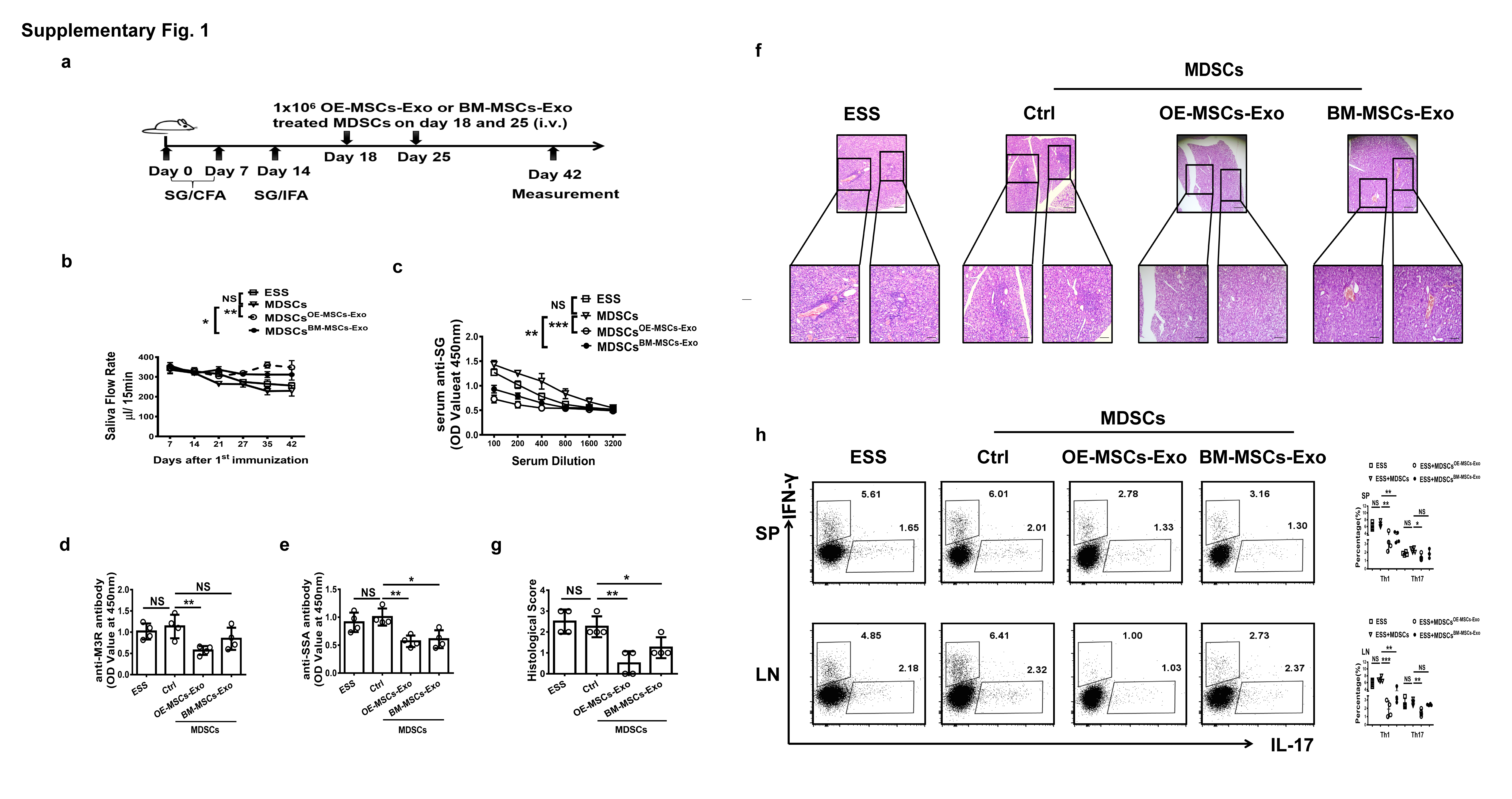

Supplement: Supplementary file 2 — Supplementary Figure 1 [file 41423_2020_587_MOESM2_ESM.tif]

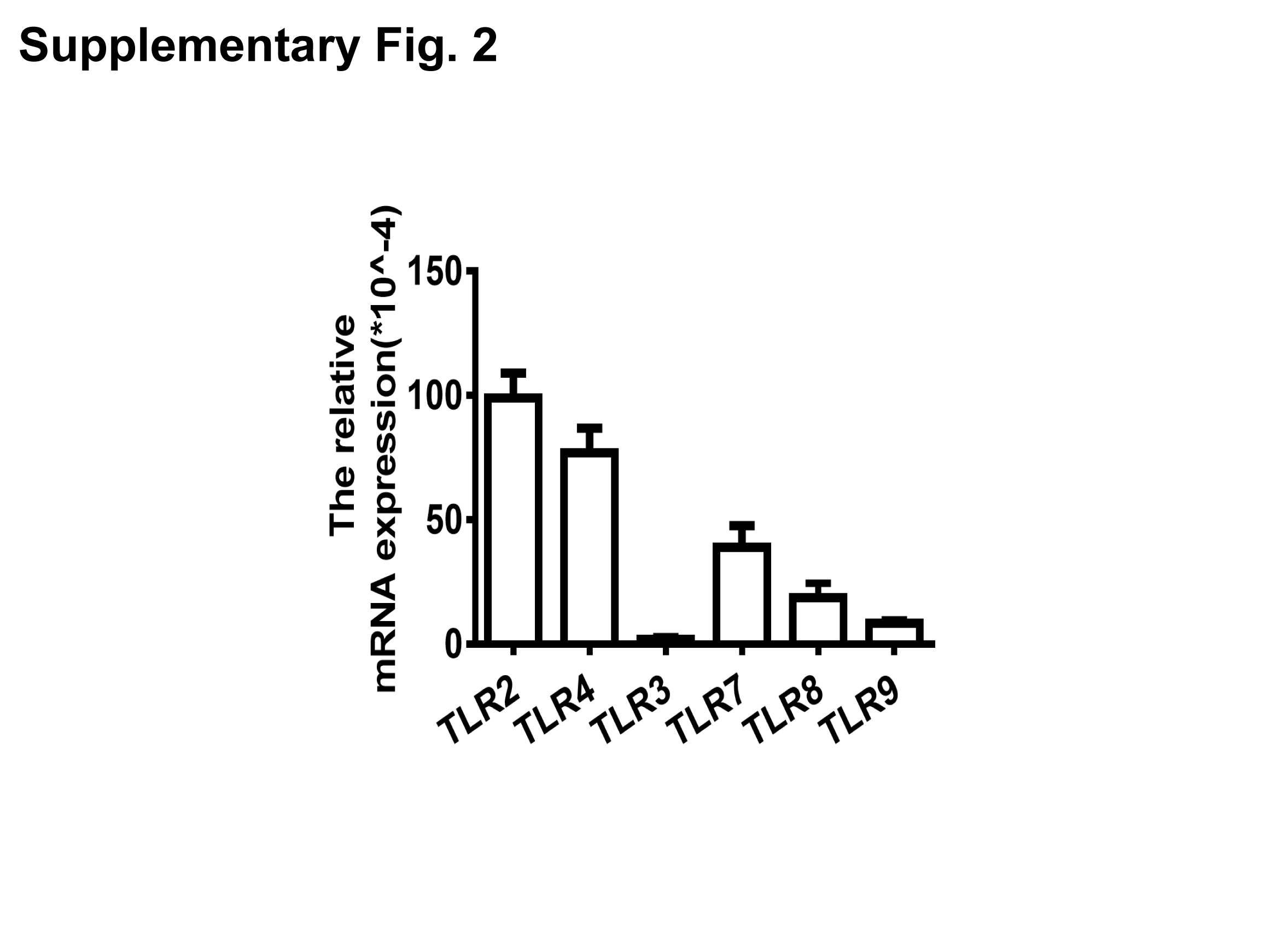

Supplement: Supplementary file 3 — Supplementary Figure 2 [file 41423_2020_587_MOESM3_ESM.tif]

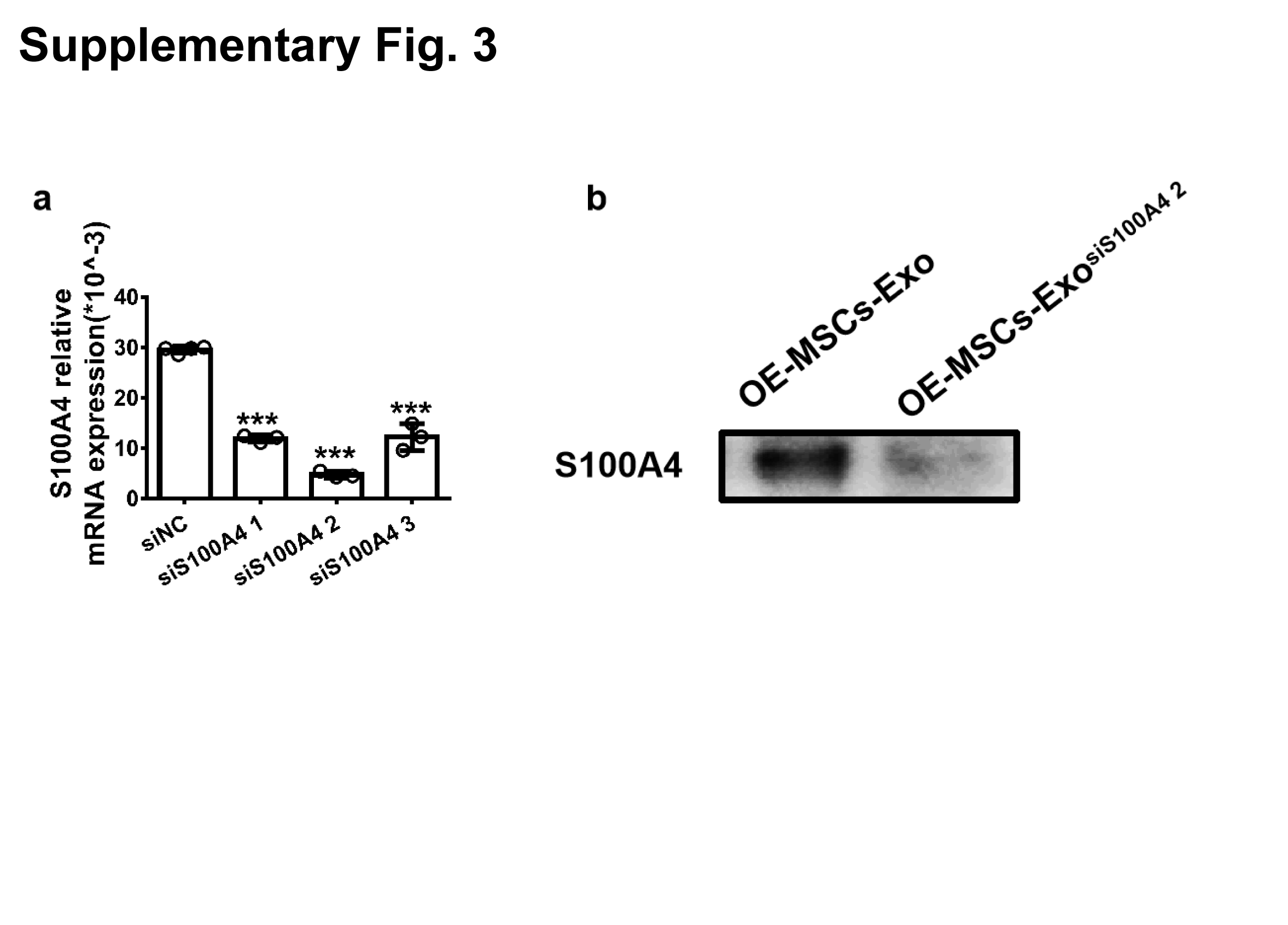

Supplement: Supplementary file 4 — Supplementary Figure 3 [file 41423_2020_587_MOESM4_ESM.tif]
